# Oxygenated Ylangene-Derived Sesquiterpenoids from the Soft Coral *Lemnalia philippinensis*

**DOI:** 10.3390/md11103735

**Published:** 2013-09-30

**Authors:** Yun-Jie Xio, Jui-Hsin Su, Bo-Wei Chen, Yen-Ju Tseng, Yang-Chang Wu, Jyh-Horng Sheu

**Affiliations:** 1 Department of Marine Biotechnology and Resources, National Sun Yat-sen University, Kaohsiung 804, Taiwan; E-Mails:yunjie0711@gmail.com (Y.-J.X.); a6152761@yahoo.com.tw (B.-W.C.); pit0424@yahoo.com.tw (Y.-J.T.); 2 Graduate Institute of Marine Biotechnology and Department of Life Science and Institute of Biotechnology, National Dong Hwa University, Pingtung 944, Taiwan; E-Mail: x2219@nmmba.gov.tw; 3 National Museum of Marine Biology and Aquarium, Pingtung 944, Taiwan; 4 College of pharmacy, China Medical University, Taichung 404, Taiwan; E-Mail: yachwu@mail.cmu.edu.tw; 5 Department of Medical Research, China Medical University Hospital, China Medical University, Taichung 404, Taiwan; 6 Graduate Institute of Natural Products, Kaohsiung Medical University, Kaohsiung 807, Taiwan

**Keywords:** *Lemnalia philippinensis*, ylangene, philippinlins A and B, cytotoxicity

## Abstract

Chemical examination of a Taiwanese soft coral *Lemnalia philippinensis* led to the isolation of three oxygenated ylangene-derived sesquiterpenoids **1**–**3**, including two new metabolites, philippinlins A and B (**1** and **2**). The structures of these compounds were elucidated on the basis of detailed spectroscopic data. Compound **1** was shown to exhibit cytotoxicity against HepG2, MDA-MB231 and A549 cancer cell lines.

## 1. Introduction

In recent years, soft corals have become one of the most prolific sources for the discovery of novel secondary metabolites [1]. Previous chemical investigations on soft corals of the genus *Lemnalia* have led to the isolation and identification of variety of ylangene-derived sesquiterpenoids [2,3,4,5]. Some of these have been found to possess various biological activities, such as cytotoxic [4] and anti-inflammatory properties [6]. Using our experience searching for new bioactive metabolites from soft corals of *Lemnalia* [7] and *Paralemnalia* [8,9,10,11], we carried out the first chemical investigation of the soft coral *Lemnalia philippinensis* with the aim of discovering interesting new metabolites. Our chemical examination of this soft coral led to the isolation of two new oxygenated ylangene-type sesquiterpenoids, philippinlins A and B (**1** and **2**), and one known compound lemnalol (**3**) [2]. The structures of **1**–**3** were established by detailed spectroscopic analysis, including extensive examination of 2D NMR (COSY, HSQC, HMBC and NOESY) correlations. The cytotoxicity of metabolites **1**–**3** towards human liver carcinoma (HepG2), human breast carcinoma (MDA-MB231) and human lung adenocarcinoma epithelial cells (A549) was evaluated, and **1** was found to be cytotoxic against the above cancer cells.

## 2. Results and Discussion

**Figure 1:**
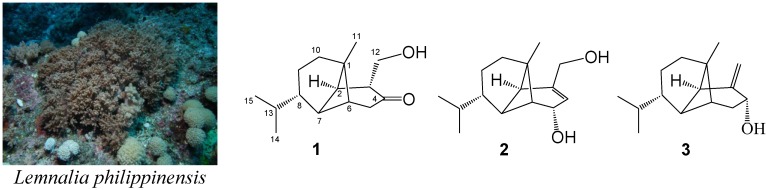
The soft coral *Lemnalia philippinensis* and the structures of philippinlins A (**1**), B (**2**), and lemnalol (**3**).

The EtOAc extract of the frozen specimen was fractionated by silica gel column chromatography and the eluted fractions were further separated utilizing normal phase HPLC to yield metabolites **1**–**3** (Figure 1). The new compounds were given the trivial names philippinlins A and B (**1** and **2**). By comparison of NMR and MS data, compound **3** was found to be identical with known lemnalol [2]. The specific optical rotation of **3** ([α]^25^_D_ −8.0) was close to that reported for lemnalol ([α]^25^_D_ −9.3) [2],which ensures that we are dealing with the same enantiomer. Compounds **1** and **2** were isolated with **3** from the same organism and should be formed by a shared biosynthetic pathway. Thus, both new compounds are suggested to possess the absolute configurations as shown in formulae **1** and **2**.

**Figure 2:**
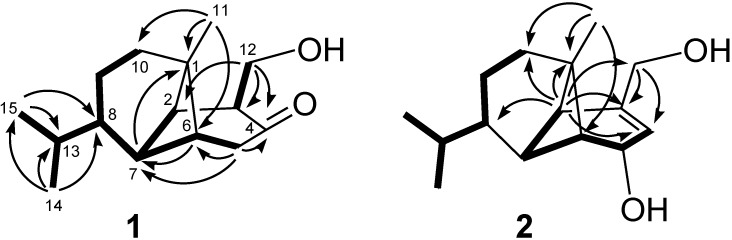
Selected COSY (▬) and HMBC (→) correlations of **1** and **2**.

**Figure 3:**
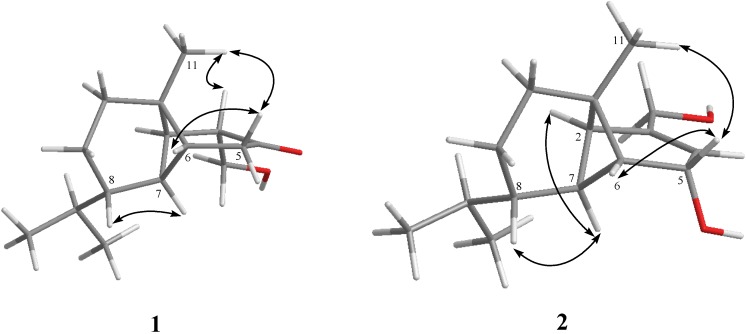
Key NOESY correlations for **1** and **2**.

Philippinlin A (**1**) was obtained as a colorless oil. The HRESIMS of **1** exhibited a pseudomolecular ion peak at *m*/*z* 259.1672 [M + Na]^+^, and established a molecular formula of C_15_H_24_O_2_, implying four degrees of unsaturation. IR absorptions were observed at 3437 and 1714 cm^−1^, suggesting the presence of hydroxy and carbonyl groups in **1**. In the ^13^C NMR and DEPT spectroscopic data (Table 1), signals of three methyls, four sp^3^ methylenes (including one oxymethylene), six sp^3^ methines, one sp^3^ quaternary carbon and one ketone were observed. From the ^1^H NMR spectrum of **1**, the signals of one oxygenated methylene (δ 3.91, dd, *J* = 10.5 and 8.5 Hz; 3.55, dd, *J* = 10.5 and 5.5 Hz) and three methyls (δ 0.90, s; 0.87, d, *J* = 7.0 Hz; 0.85, d, *J* = 7.0 Hz) were observed. The planar structure and all of the ^1^H and ^13^C chemical shifts of **1** were elucidated by 2D NMR spectroscopic analysis, in particular COSY and HMBC experiments (Figure 2). To establish the proton sequences in **1**, the ^1^H-^1^H COSY spectrum analysis established two proton sequences (Figure 2). The molecular framework of **1** was further established by an HMBC experiment, which showed the following key correlations (Figure 2): H_2_-5 to C-4, C-6 and C-7, H-6 to C-7, H-7 to C-1, H_3_-11 to C-1, C-2, C-6 and C-10, H_2_-12 to C-2, C-3 and C-4, both methyls H_3_-14 and H_3_-15 to C-8 and C-13. From above results and the chemical shifts of CH_2_-12 and C-4 (Table 1), **1** was found to possess a hydroxy group at C-12, and a ketone group at C-4. Furthermore, analysis of the ^1^H and ^13^C NMR data showed that the partial structures from C-1, C-2 and C-6 to C-15 in **1** should be identical to those of **3**. The relative configurations of all stereocenters, except C-3 and C-4 of **1**, were confirmed to be the same as those of **3** by comparison of the proton shifts and NOE correlations (Figure 3). One hydroxymethyl group at C-3 was assigned the *α*-configuration primarily due to the NOE correlation between H-3 and H_3_-11. Thus, the structure of sesquiterpenoid **1** was established.

**Table 1:** ^13^C and ^1^H NMR spectral data for compounds **1** and **2.**

C/H	1		2	
	δ_c_ (Mult.) ^a^	δ_H_ (*J* in Hz) ^b^	δ_c_ (Mult.) ^c^	δ_H_ (*J* in Hz) ^d^
1	41.6 (C)		48.9 (C)	
2	37.3 (CH)	2.07 dd (7.0, 2.0)	40.9 (CH)	2.24 d (6.0)
3	51.5 (CH)	2.74 ddd	150.3 (C)	
		(8.5, 5.5, 2.0)		
4	216.7 (C)		119.7 (CH)	5.66 brs
5	43.5 (CH_2_)	α: 2.69 dd (19.0, 1.5)	70.2 (CH)	4.36 brs
		β: 2.52 dd (19.0, 3.5)		
6	46.2 (CH)	1.70 m	54.8 (CH)	1.82 m
7	42.8 (CH)	1.74 m	42.3 (CH)	1.89 m
8	44.2 (CH)	1.65 m	44.9 (CH)	1.60 m
9	21.6 (CH_2_)	1.70 m; 1.59 m	21.9 (CH_2_)	1.67 m; 1.59 m
10	36.3 (CH_2_)	1.79 m; 1.71 m	36.8 (CH_2_)	1.73 m
11	18.2 (CH_3_)	0.90 s	19.1 (CH_3_)	0.82 s
12	63.4 (CH_2_)	3.91 dd (10.5, 8.5)	65.4 (CH_2_)	4.06 s
		3.55 dd (10.5, 5.5)		
13	32.4 (CH)	1.50 m	32.5 (CH)	1.58 m
14	19.8 (CH_3_)	0.85 d (7.0)	19.4 (CH_3_)	0.87 d (6.4)
15	19.5 (CH_3_)	0.87 d (7.0)	20.0 (CH_3_)	0.86 d (6.4)

Spectra recorded at *^a^* 125 MHz in CDCl_3_; *^b^* 500 MHz in CDCl_3_; *^c^* 100 MHz in CDCl_3_; *^d^* 400 MHz in CDCl_3_.

Philippinlin B (**2**) was obtained as a white powder and showed a [M + Na]^+^ ion peak in the HRESIMS spectrum corresponding to the molecular formula C_15_H_24_O_2_, the same as that of **1**. IR absorption was observed at 3358 cm^−1^, again suggesting the presence of the hydroxy group in **2**. Also, unlike **1**, the absence of carbonyl absorption was shown in the IR spectrum. The planar structure and all of the ^1^H and ^13^C chemical shifts of **2** were elucidated by 2D NMR spectroscopic analysis, in particular COSY and HMBC experiments (Figure 2). Thus, **2** was found to possess one double bond at C-3/C-4, two hydroxy groups at C-5 and C-12. The relative configurations of the six chiral centers at C-1, C-2, C-5, C-6, C-7 and C-8 in **2** were elucidated by detailed NOE analysis, as shown in Figure 2. In these experiments, it was found that H-5 showed NOE interactions with H-6 and H_3_-11. Thus, assuming the *β*-orientation of H-5, both H-6 and H_3_-11 should be positioned on the *β* face. On the basis of the above findings and detailed examination of other NOE correlations (Figure 3), the relative structure of compound **2** was determined.

The cytotoxicity of metabolites **1**–**3** against the growth of HepG2, MDA-MB231 and A549 carcinoma cells was studied. Compound **1** showed cytotoxicity towards HepG2, MDA-MB231, and A549 cancer cell lines with IC_50_ values of 16.0, 16.3, and 15.8 μg/mL, respectively. While lemnalol has been shown to possess remarkable anti-inflammatory activities [6,12,13], compounds **2** and **3** did not exhibit cytotoxicity towards the above cancer cell lines.

## 3. Experimental Section

### 3.1. General Experimental Procedures

Optical rotation values were measured with a Jasco-P1010 digital polarimeter. Infrared spectra were obtained on a Varian Diglab FTS 1000 FT-IR spectrophotometer. NMR spectra were recorded on a Varian Mercury Plus 400 FT-NMR at 400 MHz for ^1^H and 100 MHz for ^13^C in CDCl_3_or C_5_D_5_N at 25 °C. ESIMS and HRESIMS data were recorded on a Bruker APEX II mass spectrometer. Column chromatography was performed on silica gel (230–400 mesh, Merck, Darmstadt, Germany). TLC was carried out on precoated Kieselgel 60 F_254_ (0.25 mm, Merck, Darmstadt, Germany) and spots were visualized by spraying with 10% H_2_SO_4_ solution followed by heating. Normal phase HPLC (NP-HPLC) was performed using a system comprised of a Hitachi L-7110 pump, a Hitachi L-7455 photodiode array detector and a Rheodyne 7725 injection port. A normal phase column (Supelco Ascentis^®^ Si Cat #:581515-U, 25 cm × 21.2 mm, 5 μm) was used for NP-HPLC. Reverse phase HPLC (RP-HPLC) was performed using a system comprised of a Hitachi L-7100 pump, a Hitachi L-2455 photodiode array detector and a Rheodyne 7725 injection port. A reverse phase column (Varian Polaris C18-A, 250 mm × 10 mm, 5 μm) was used for RP-HPLC.

### 3.2. Animal Material

*L. philippinensis*, taxonomically identified by Prof. Chang-Feng Dai of National Taiwan University, was collected by hand using scuba off the coast of Lanyu, Taiwan, in August 2008, at a depth of 10–15 m, and stored in a freezer until extraction. A voucher sample was deposited at the Department of Marine Biotechnology and Resources, National Sun Yat-sen University*.*

### 3.3. Extraction and Isolation

Sliced tissues of the soft coral *L. philippinensis* (0.8 kg, wet wt) were exhaustively extracted with EtOAc. The combined EtOAc extract was concentrated under reduced pressure. The EtOAc extract was evaporated to yield a residue (10.7 g), which was subjected to open column chromatography on silica gel stepwisely eluting with *n*-hexane-EtOAc mixture and EtOAc-MeOH mixture, to give 25 fractions. Fraction 7, eluted with *n*-hexane–EtOAc (20:1), was further separated by Sephadex LH-20 column chromatography with acetone as eluent to yield 5 subfractions (7A–E). Subfraction 7D was separated by silica gel open column chromatography with gradient elution (*n*-hexane–CH_2_Cl_2_, 4:1) to afford **3** (156 mg) and subfraction 7E was also separated by normal phase HPLC using *n*-hexane–CH_2_Cl_2_ (4:1) to afford **1** (3.9 mg). The combined fractions 18 and 19, eluted with *n*-hexane–EtOAc (1:1–1:2), were separated by silica gel open column chromatography with gradient elution (*n*-hexane–EtOAc, 7:2) to yield 8 subfractions (18A–H). Subfraction 18G was separated by normal phase HPLC using *n*-hexane–EtOAc (2:1) to afford **2** (1.1 mg).

Philippinlin A (**1**): colorless oil; [α]^25^_D_ = −97 (*c* 0.3, CHCl_3_); IR (neat) ν_max_ 3437, 2941, 1714, 1459 and 1376 cm^−1^; ^1^H and ^13^C NMR data, see Table 1; ESIMS *m*/*z* 259 [M + Na]^+^; HRESIMS *m*/*z* 259.1672 (calcd. for C_15_H_24_O_2_Na, 259.1674).

Philippinlin B (**2**): white powder; mp 92–93 °C; [α]^25^_D_ = +39 (c 0.1, CHCl_3_); IR (neat) ν_max_ 3358, 2932, 2862 and 1464 cm^−1^; ^1^H and ^13^C NMR data, see Table 1; ESIMS *m*/*z* 259 [M + Na]^+^; HRESIMS *m*/*z* 259.1672 (calcd. for C_15_H_24_O_2_Na, 259.1674).

### 3.4. Cytotoxicity Testing

Cell lines were purchased from the American Type Culture Collection (ATCC). Cytotoxicity assays of compounds **1**–**3** were performed using the MTT [3-(4,5-dimethylthiazol-2-yl)-2,5-diphenyltetrazolium bromide] colorimetric method [14,15]. Doxorubicin, employed as positive control, exhibited cytotoxic activity toward HepG2, MDA-MB231 and A549 cancer cell lines with IC_50_’s 0.4, 1.4, and 1.6 μg/mL, respectively.

## 4. Conclusions

Two new oxygenated ylangene-derived compounds, philippinlins A (**1**) and B (**2**) and a known compound lemnalol (**3**), were discovered from the soft coral *L*. *philippinensis*. Compound **1** was found to exhibit cytotoxicity towards HepG2, MDA-MB231 and A549 cancer cell lines.

## Supplementary Files

Supplementary File 1 Supplementary Information (PDF, 424 KB)

Supplementary File 2 3D structure (MOL, 2 KB)

Supplementary File 3 MOL-Document (MOL, 2 KB)

Supplementary File 4 3D structure (MOL, 2 KB)

Supplementary File 5 MOL-Document (MOL, 2 KB)

Supplementary File 6 3D structure (MOL, 2 KB)

Supplementary File 7 MOL-Document (MOL, 2 KB)
