# Supplementary material for: Oxygenated Ylangene-Derived Sesquiterpenoids from the Soft Coral Lemnalia philippinensis
Source: Mar Drugs. 2013 Sep 30;11(10):3735–41. doi: 10.3390/md11103735 (PMC3826132; doi:10.3390/md11103735)

Supplementary Information

Figure S1. ^{1}H NMR spectrum of **1** in CDCl3 at 500 MHz.

Figure S2. ^{1}H NMR spectrum (2.4–4.0 ppm) of **1** in CDCl3 at 500 MHz.

Figure S3. ^{13}C NMR spectrum of **1** in CDCl3 at 125 MHz.

Figure S4. COSY spectrum of **1** in CDCl3.

Figure S5. HMBC spectrum of **1** in CDCl3.

Figure S6. ^{1}H NMR spectrum of **2** in CDCl3 at 400 MHz.

Figure S7. ^{13}C NMR spectrum of **2** in CDCl3 at 100 MHz.

Figure S8. COSY spectrum of **2** in CDCl3.

Figure S9. HMBC spectrum of **2** in CDCl3.

Figure S1. ^{1}H NMR spectrum of **1** in CDCl3 at 500 MHz.

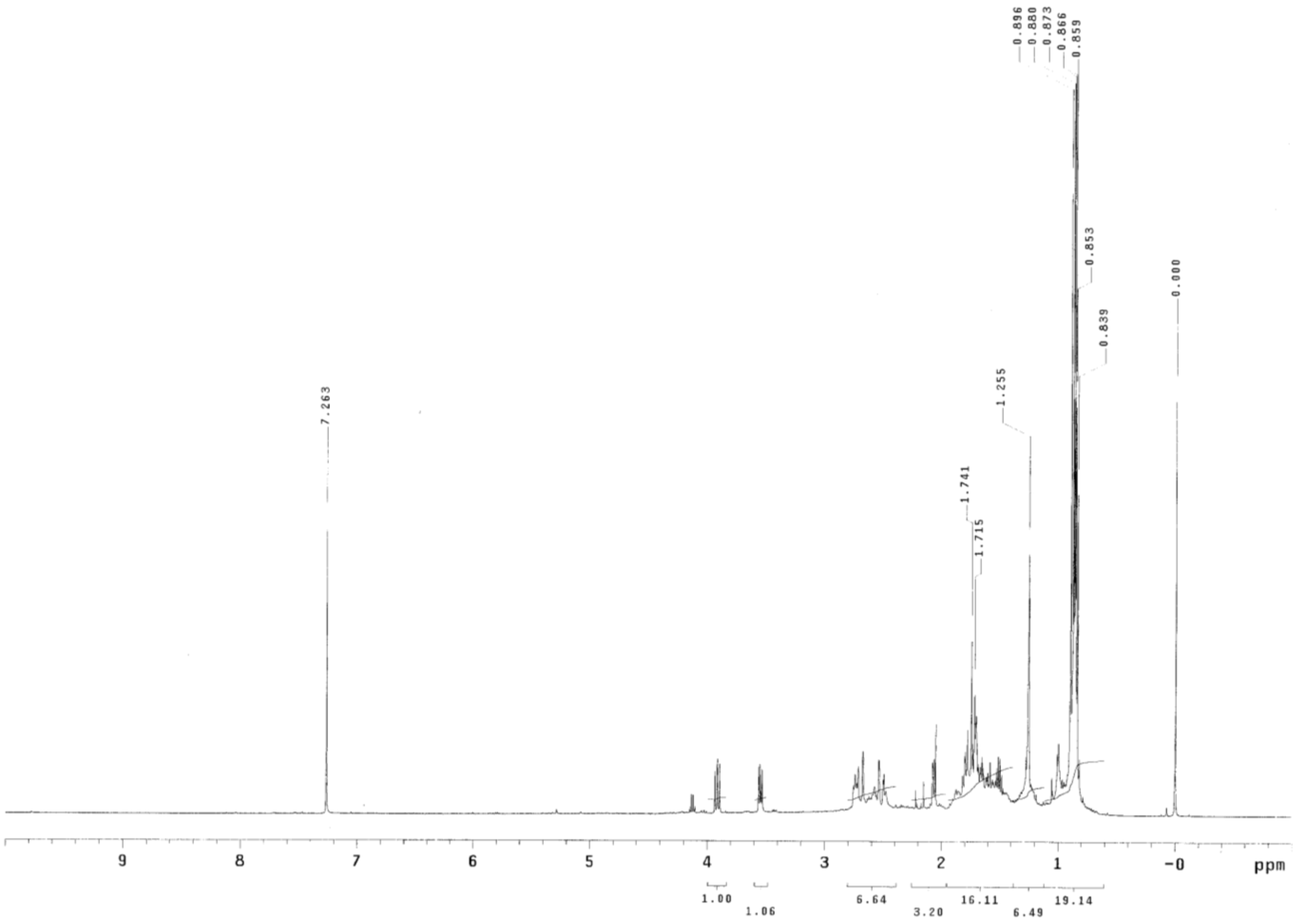

Figure S2. ^{1}H NMR spectrum (2.4–4.0 ppm) of **1** in CDCl3 at 500 MHz.

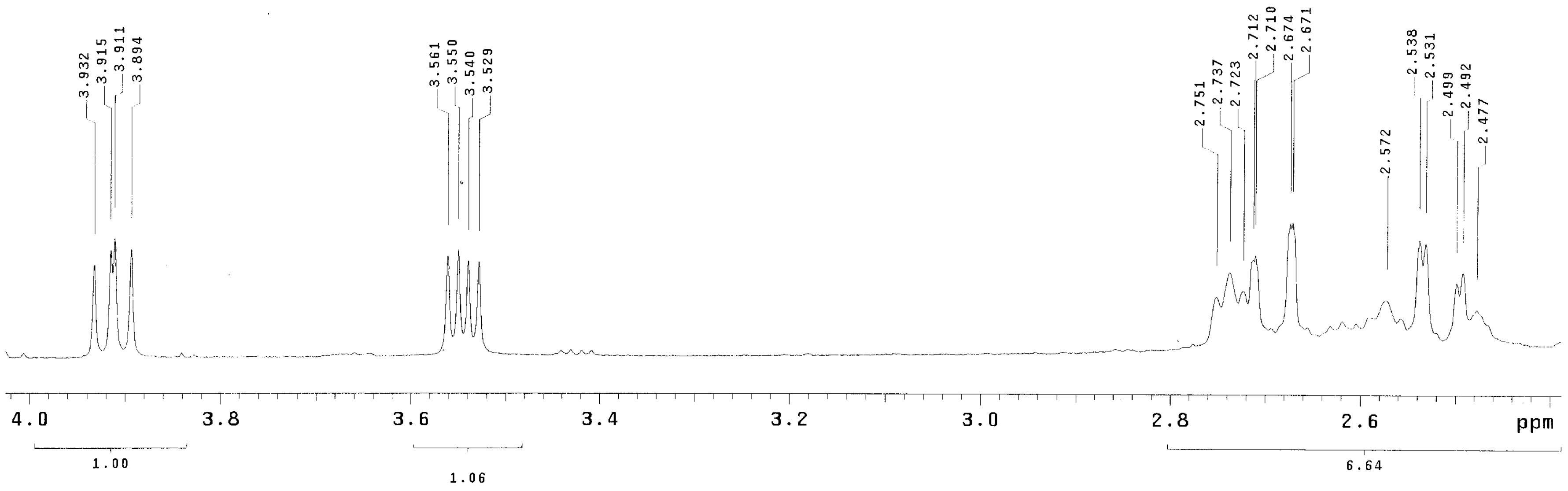

Figure S3. ^{13}C NMR spectrum of **1** in CDCl3 at 125 MHz.

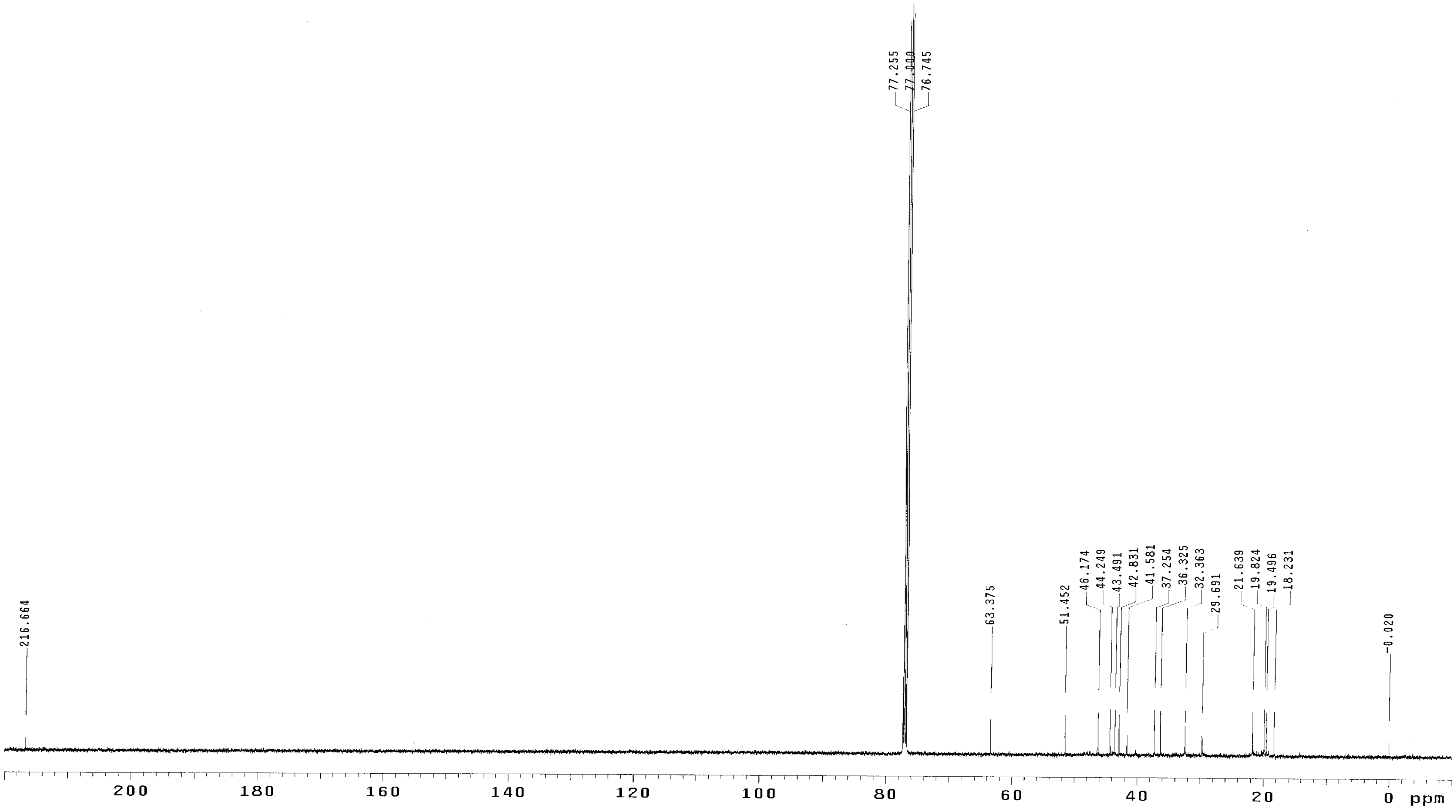

Figure S4. COSY spectrum of **1** in CDCl3.

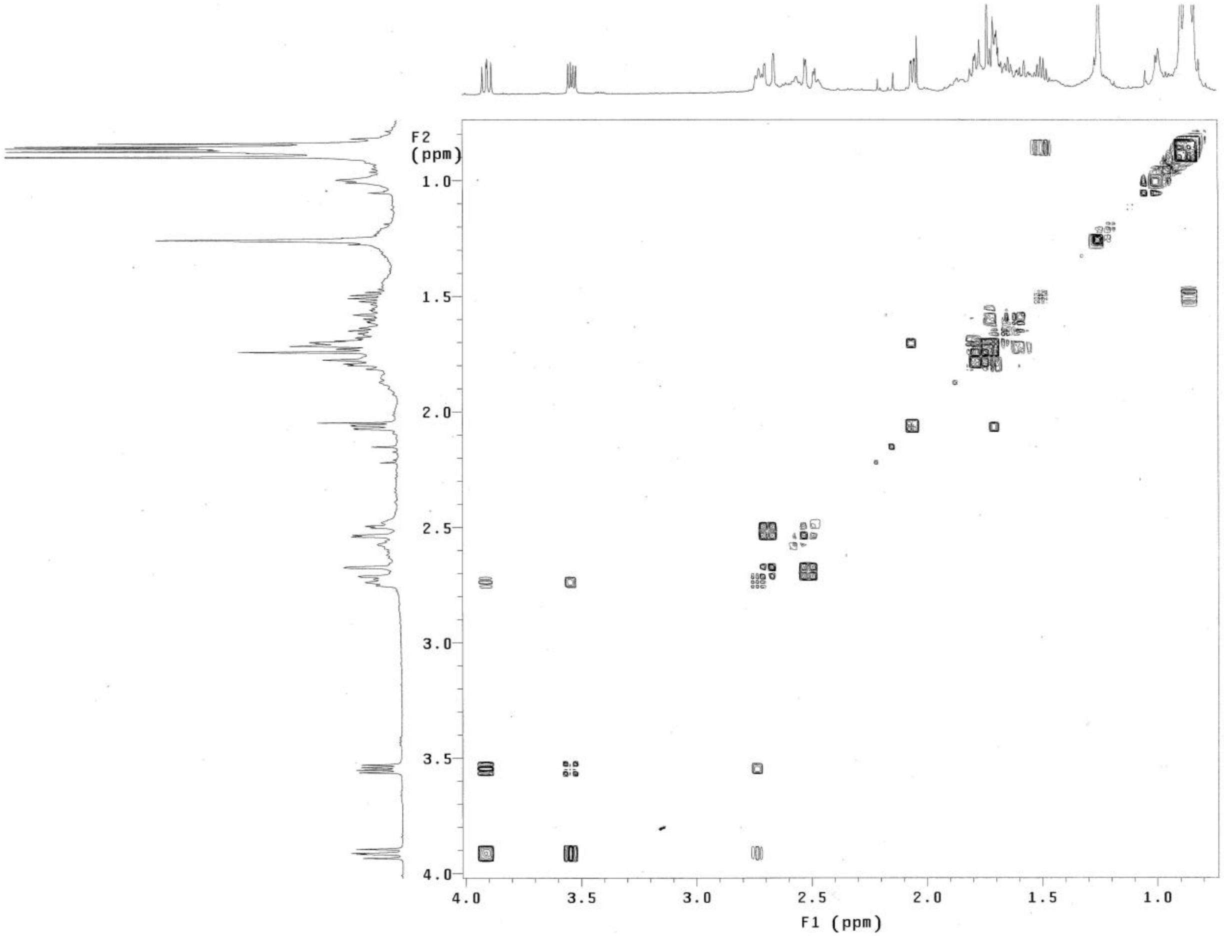

Figure S5. HMBC spectrum of **1** in CDCl3.

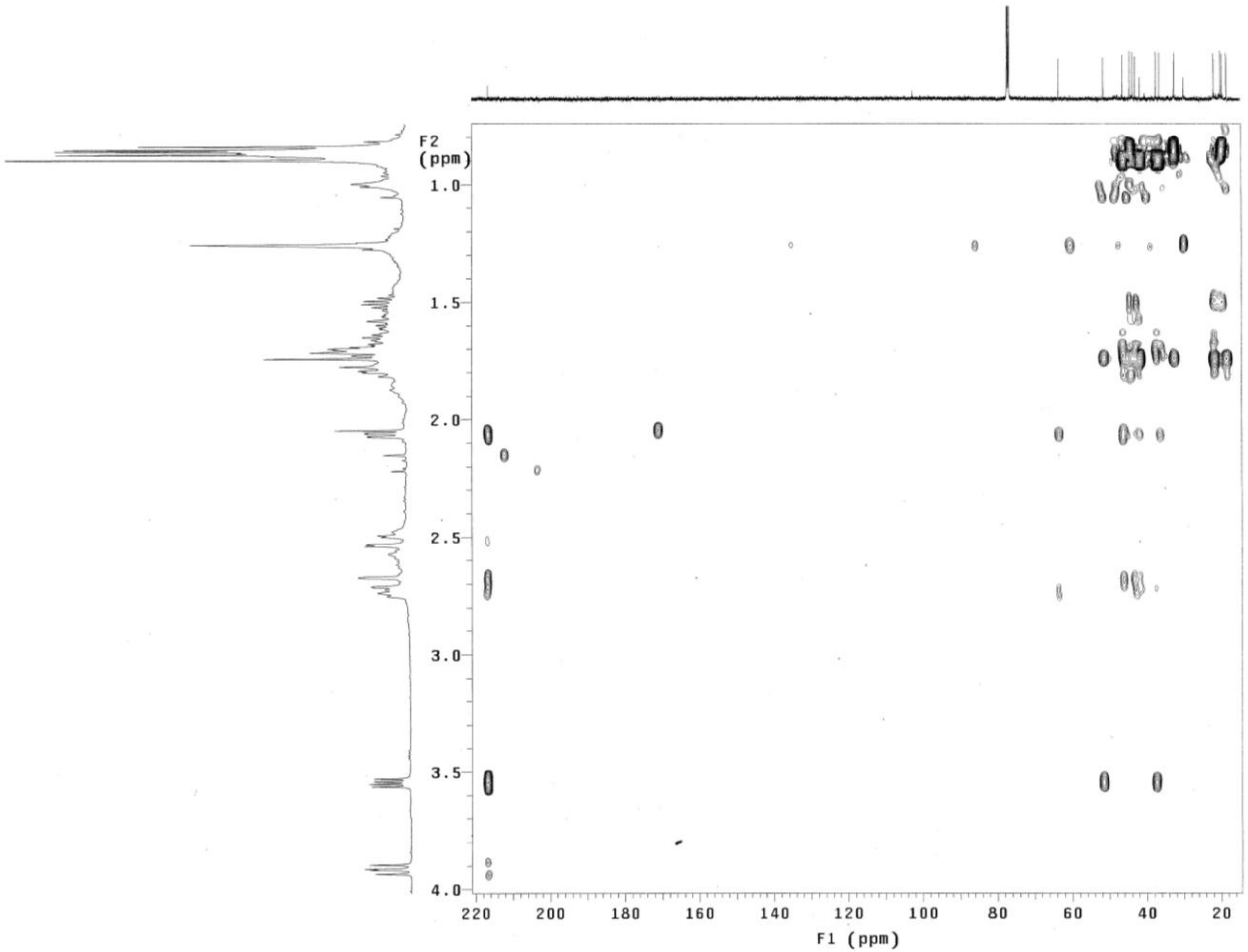

Figure S6. ^{1}H NMR spectrum of **2** in CDCl3 at 400 MHz.

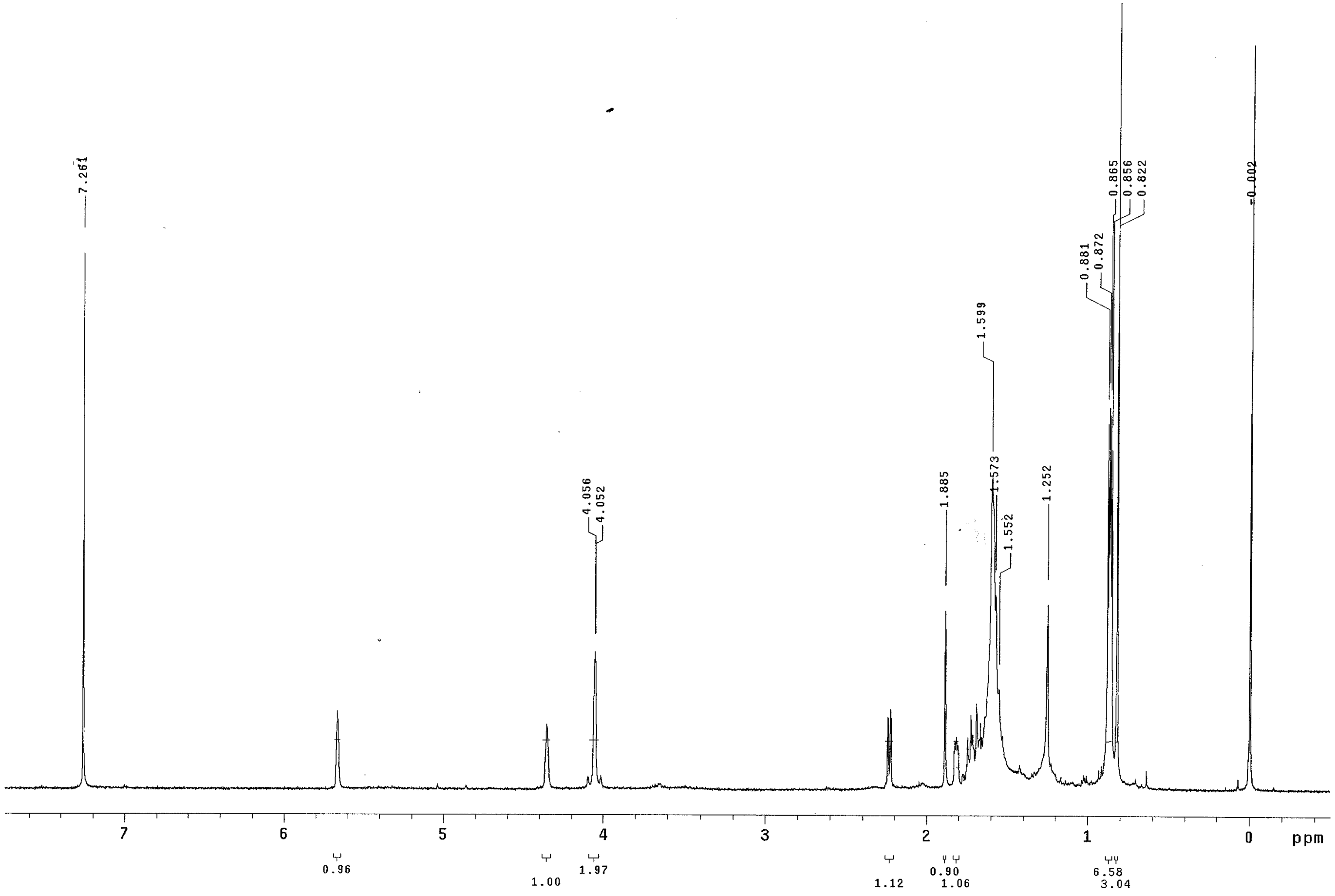

Figure S7. ^{13}C NMR spectrum of **2** in CDCl3 at 100 MHz.

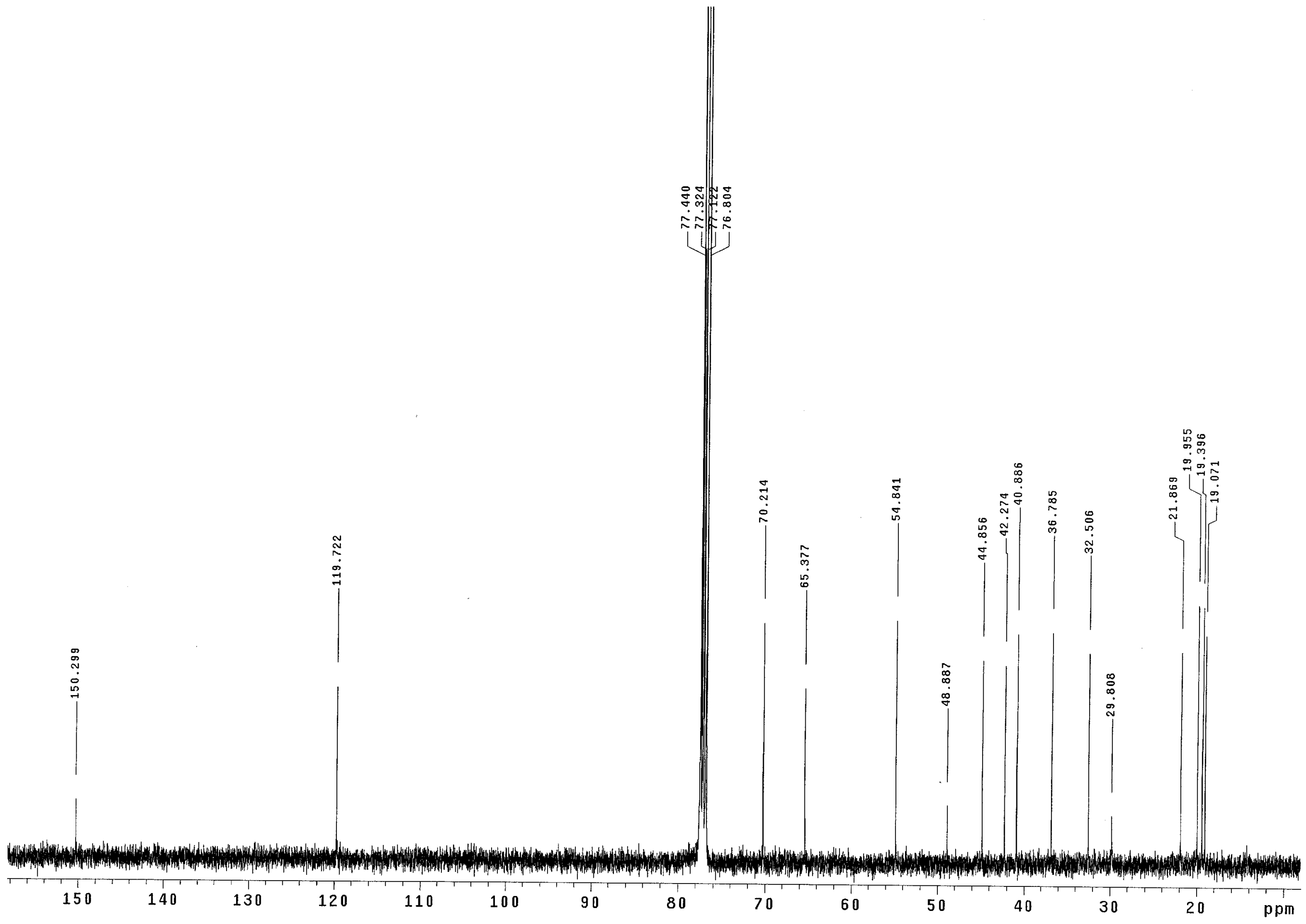

Figure S8. COSY spectrum of **2** in CDCl3.

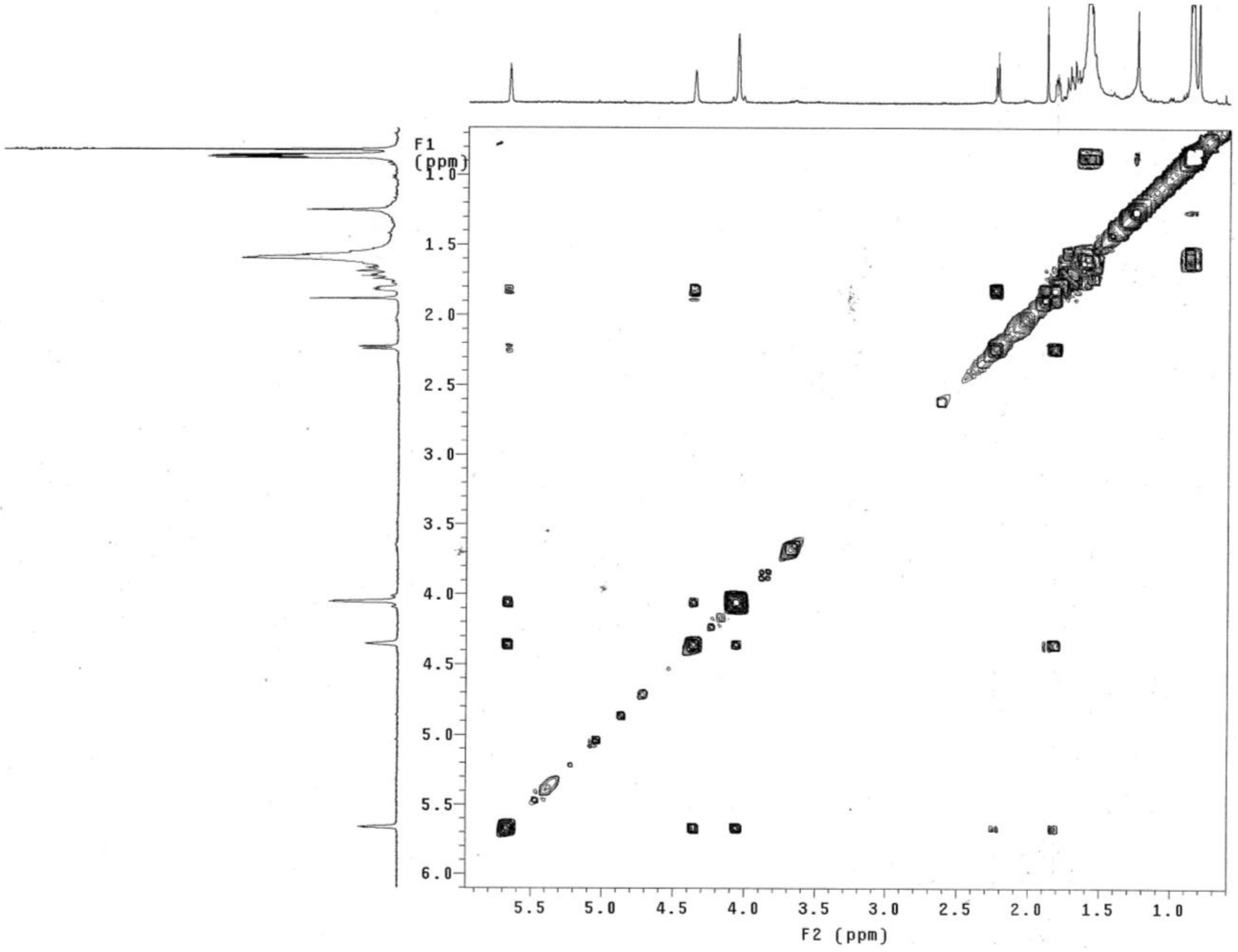

Figure S9. HMBC spectrum of **2** in CDCl3.

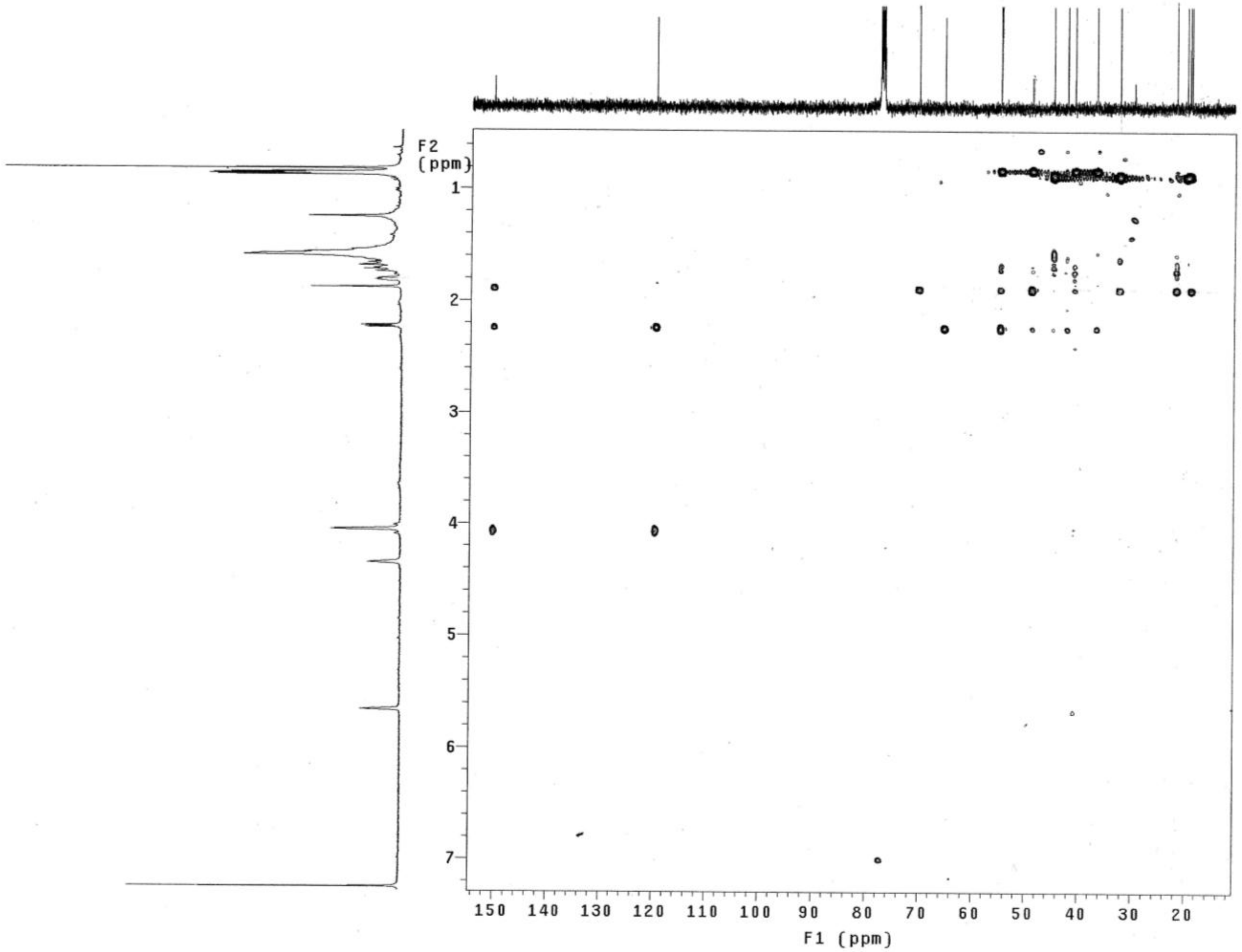

Supplement: Supplementary File 1 — Supplementary Information (PDF, 424 KB) [file marinedrugs-11-03735-s001.pdf]
